# Does Varicocelectomy Improve Gonadal Function in Men with Hypogonadism and Infertility? Analysis of a Prospective Study

**DOI:** 10.1155/2011/916380

**Published:** 2011-11-29

**Authors:** Vasan Sathya Srini, Srinivas Belur Veerachari

**Affiliations:** Department of Andrology, Ankur Health Care Private Limited, No. 55, 20th Main, 1st Block, Rajajinagar, Bengaluru 560010, India

## Abstract

Varicocele in infertile males is associated with Leydig cell dysfunction and hypogonadism. The effect of varicocelectomy on serum testosterone level is not yet established. We analysed 200 heterosexual infertile men diagnosed to have clinical varicocele they were divided into two groups: group 1 (100 men) had microsurgical varicocelectomy, and group 2 (100 patients) underwent assisted reproduction procedures. All participants had semen analysis, serum levels of follicle-stimulating hormone (FSH), luteinizing hormone (LH), prolactin, and total testosterone (TT), measured both at recruitment time and 6 months later. In group 1, the mean TT level increased significantly after varicocelectomy (1.644 ± 0.029 to 2.461 ± 0.0198 ng/dL, *P* < 0.0001) and testicular size correlated with the mean change in TT (*P* = 0.001). No similar change was found in group 2. Out of the 100 patients in group 1, 78 had postoperative normalization of TT unlike only 16 men in group 2.

## 1. Introduction

The prevalence of a varicocele among the general male population is about 15%, whereas it is reported that 19% to 41% of infertile men have a palpable varicocele [1]. Theories related to the negative effect of varicocele on testicular functions include testicular hyperthermia, hormonal dysfunctions, increased testicular blood flow due to venous engorgement, reflux of renal or perirenal metabolites, and hypoxia [2–4]. Weiss et al. [5] reported impaired testosterone synthesis in patients with varicocele. Other studies have suggested Leydig cell dysfunction and impaired testosterone synthesis [6, 7]. The effect of varicocelectomy on serum testosterone level is not yet fully established. While some studies found no significant effect, others reported a normalization of the testosterone level following surgical repair of the varicocele [7–12]. In a study conducted by Gat et al., it was concluded that varicocele is a bilateral disease and the damage to the testicular tissue was hypoxia which deteriorates sperm production and testosterone production due to abnormal arterial flow of oxygen to the sperm production site (Seminiferous tubules) and the testosterone production site Leydig cells. It is caused due to the destruction of the one-way valve mechanism in the internal spermatic veins which leads to increase in hydrostatic pressure in the testicular venous drainage system exceeding the pressure of the testicular arterial system. This is a unique biological phenomena that exists in the bipedal, human species due to the erect posture [10].

Normal serum testosterone is essential in maintaining peripheral autonomic and sensory nerve structure [13] as well as the function of many pelvic ganglion neurons [14]. Androgen plays an important role in regulating nitric oxide synthase isoforms in penile tissue [15–17]. Testosterone is also important for erectile function, as the trabecular smooth muscle is regulated for detumescence and erection [18].

There are very few studies in the literature analyzing androgen deficiency in infertile men with varicocele and the impact of varicocelectomy on hypogonadal status. Hence, we designed a prospective nonrandomized comparative study with the aim to investigate the effect of varicocelectomy on serum total testosterone (TT) level.

## 2. Materials and Methods

We included 200 heterosexual infertile men diagnosed to have clinical varicocele associated with primary or secondary infertility with a serum testosterone level < 280 ng/dL. All men had been in a stable sexual relationship for at least 12 months. The varicocele was detected by physical examination and confirmed by color flow Doppler ultrasound examination. All participants had semen analysis, serum levels of follicle stimulating hormone (FSH), luteinizing hormone (LH), prolactin, and total testosterone (TT), measured both at recruitment time, 6 and 12 months later. All of them signed an informed consent to be enrolled for the study, and institutional review board approval was obtained before the start of the study which was nonrandomized prospective controlled study.

### 2.1. Exclusive Criteria

They excluded participants with history of undescended testis, mumps orchitis, hypogonadotropic hypogonadism, hyperprolactinemia, use of testosterone replacement therapy and/or antiestrogen, aromatase enzyme inhibitors, morbid obesity with body mass index (BMI) ≥40 kg/m^2^, uncontrolled diabetes (glycated hemoglobin > 7%) or uncontrolled hypertension (blood pressure ≥ 140/90) after chemotherapy, after radiotherapy, and after orchidectomy.

### 2.2. Intervention

In addition to routine medical history taking, general and genital examination, all participants underwent semen analysis according to the World Health Organization guidelines [19]. Serum TT was measured in the morning using the electrochemiluminescence immunoassay (ECLIA) used on the Roche Elecsys 2010 (Hitachi, Japan) (normal range 280–800 ng/dL). Serum FSH, LH, and prolactin were also measured using the ECLIA, Roche, Japan. The normal ranges are 1.5–12.4 mIU/mL, 1.7–8.6 mIU/mL, and 4.6–21.4 ng/dL, respectively. 

Scrotal sonography was performed with the participant in the supine position. Examinations were performed with high-resolution ultrasound transducers (7.5 MHz) linear arrays, which were appropriate for all participants. Gray-scale imaging was used to determine the volume of the testis ([length × width × thickness] × 0.52). Maximum vein diameter was measured. Color Doppler was then used to detect the reversal of blood flow in the veins [20]. In cases with bilateral varicocele, the average vein diameter was used for statistical correlations. 

Couples were counseled about their case, and the exact nature of the problem was explained. They were asked to choose between microsurgical varicocelectomy or in vitro fertilization (IVF)/intracytoplasmic sperm injection (ICSI) attempts. The nature of each approach was discussed with the participants.

The microsurgical varicocelectomy procedure was performed as described by Zini et al. [21]. Both external and internal spermatic veins were ligated and divided, while the testicular artery and lymphatic vessels were spared. 

Participants were asked to revisit the centre after 6 and 12 months. A new semen analysis was performed, and a morning blood sample was withdrawn for the measurements of TT, FSH, and LH.

### 2.3. Statistical Analysis

Descriptive statistics (mean ± standard deviation, as well as frequencies and percentages) were computed for all clinical and demographic variables. Median (quartiles) expressed data were not distributed. Comparisons between the two groups were carried out using an unpaired Student's *t*-test. Based on Kolmogorov-Smirnov test used to test the parameter distribution normality, if the parameters are not normally distributed, nonparametric tests were used and are mentioned when used below the tables. Chi-square was used for contingency table analysis; Pearson's correlation coefficients were computed to determine the strength and form of associations. For non-normally distributed data Spearman's methods were used. All analysis was conducted using SPSS 8.0 for Windows (SPSS Inc., Chicago, Ill, USA). Significance determined at 0.05 was used throughout all statistical tests. All *P* values reported in this paper are two-tailed unless stated otherwise.

## 3. Results

We included 200 heterosexual infertile men diagnosed to have varicocele. They were divided into two groups: group 1 (study group) underwent microsurgical varicocelectomy, and group 2 (control group) opted for assisted reproduction techniques (ART). The first hundred patients choosen for either group were included in the study. One hundred forty-nine had primary infertility, while fifty-one had secondary infertility. 

The distributions of patients in terms of age group were almost similar. The age ranged from 24 years to 49 years. The mean age was 30.04 ± 4.9 years in the study group and 31.73 ± 4.5 years in the control group (Table 1). The infertility duration in study group was 3.23 ± 1.75 years and in control group was 2.39 ± 0.98 years (Table 6). 

In study group, 34 (17%) participants had bilateral varicocele and 66 (33%) had left varicocele. In control group, 36 (18%) participants had bilateral varicocele and 64 (32%) had left varicocele (Table 2). 

The average testosterone levels in the study group was 177.2 ± 18.44 ng/dL and the average testosterone levels in the control group were 184.52 ± 10.60 ng/dL (Table 3). 

The average testes sizes were 8.14 ± 4.26 mL and 8.76 ± 1.99 mL for study and control groups, respectively (*P* = 0.181) (Table 4). 

The average internal spermatic vein diameters were 3.79 ± 0.42 mm and 3.52 ± 0.37 for study and control groups, respectively (*P* = 0.132) (Table 5). 

Decreased sexual desire was seen in 76 participants of study group and in 68 participants of control group. Premature ejaculation was observed in 19 in the study group and in 12 in control group. There were nonsignificant differences in clinical parameters, semen parameters, FSH, LH, and prolactin levels between men who underwent varicocelectomy and those who opted for ART. 

In study group, TT rosed significantly from 1.77 ± 0.18 ng/mL before varicocelectomy to 3.01 ± 0.43 ng/mL after 12 months of follow-up postsurgery (Table 7). There was a significant positive correlation between the mean change in TT in this group Δ + 1.240 (*P* < 0.001). This was associated with an insignificant drop in serum LH and serum FSH levels. In control group, nonsignificant reduction was seen during the follow-up period of 6 months. The vein diameter did not correlate with the change in the TT values. However, a significant correlation was found with the mean testicular size change from 8.15 ± 3.49 cc to 9.65 ± 3.51 cc, and with change of Δ + 1.508 and change in the TT values Δ + 1.240 (*P* < 0.001) before and after surgery. 

The mean change in TT in men who underwent bilateral varicocelectomy was from 1.75 ± 0.18 ng/mL to 2.93 ± 0.29 ng/mL, with change of Δ + 1.18 was found to be lower than those who underwent left varicocelectomy in whom the mean change in TT was from 1.78 ± 0.16 ng/mL to 3.05 ± 0.40 ng/mL, with change of Δ + 1.27, the difference however was found to be insignificant. 

There was also significant improvement in sperm count from 12.18 ± 5.53 millions/mL to 17.43 ± 6.17 millions/mL (*P* < 0.001) and the percent of progressively motile sperm. There was no significant change in sperm volume, viability, or percent of abnormally formed sperm after surgery. 

In the study group, 44 of the participants suffered from ED before varicocelectomy, as compared with 31 after 12 months of surgery (*P* = 0.094). However, in the control group, there was an increase from 39 to 41 in the participants who suffered ED during the follow-up period. This was an incidental isolated finding of this study. 

## 4. Discussion

The serum testosterone level showed significant increase after varicocelectomy unlike the changes in the control group in our study. This is in agreement with the previous work of other authors [8–10]. Su et al. [8] reported that serum TT, but not FSH or LH levels, increased following microsurgical varicocelectomy. The increase in serum TT was greater in cases of bilateral varicocelectomy as compared with unilateral cases [8]. In our study, we failed to demonstrate such differences. Hurtado de Catalfo et al. [9] also reported normalization of testosterone levels in patients who underwent microsurgical varicocelectomy. Gat et al. [10] reported significant increases in serum TT and free testosterone following spermatic vein embolization not related to the varicocele clinical grade. Su et al. [8] also reported no significant correlation between varicocelectomy clinical grade and postvaricocelectomy improvement in serum TT. Pasqualotto et al. [22] reported that postoperative serum TT increases were limited to patients who had more than 10 veins ligated during varicocelectomy. The clinical grade and the vein diameter might not be the determining factors for how varicocele affects testicular functions. Shiraishi et al. [23] showed that scrotal temperature is related to the deterioration in sperm density, sperm motility, and serum FSH and testosterone levels irrespective of the varicocele grade. 

Serum testosterone increased after varicocelectomy in the study group. However, the magnitude of this increase was not the same in all participants. The same observation had been documented by other investigators [11, 12]. These findings suggest that improved spermatogenesis might have a mechanism other than increased testosterone production. Moreover, classification of men based on their preoperative TT may explain variation in TT improvement between different studies. Many studies that failed to demonstrate the positive change in the serum testosterone level following varicocelectomy did not characterize patients with low preoperative serum testosterone. Reşorlu et al. [24] reported no changes in serum TT levels following varicocelectomy in 96 patients treated for infertility. Our results are in agreement with Su et al. [8] in that they observed an inverse correlation between preoperative testosterone levels and changes in testosterone levels after varicocelectomy [8]; however, in our study, we were unable to demonstrate the same. This suggests that varicocelectomy can reverse the Leydig cell dysfunction induced by varicocele in certain patients. Rajfer et al. [25] reported decreased testicular testosterone following induction of varicocele in rats. This is confirmed by Ghosh and York in their study [26]. The mechanism by which testosterone synthesis is affected is not yet clear. Possible mechanisms include reduced activity of 17,20-desmolase and 17-alpha-hydroxylase enzymes as a result of testicular hyperthermia [27] and impaired Leydig cell response to gonadotrophin stimulation [28, 29]. 

In this current study, serum LH and serum FSH decreased insignificantly following varicocelectomy in the hypogonadal patients (study group), while there was no significant change in the control group; this might be attributed to improvement in Leydig cell function, which was increased due to raise in serum TT in the study group. Previous researchers reported no effect of varicocelectomy on serum LH, even when mean serum TT levels improved [8]. This might be due to the fact that eugonadal and hypogonadal patients were mixed together in the patient population. 

It was also noticed that the study group in whom varicocelectomy was done noticed improved erectile function postoperatively. This might be related to improvement in the TT level which plays an important role in the male sexual response and regulates the timing of the erectile process as a function of sexual desire, thereby coordinating penile erection with sex [30]. After varicocelectomy, we observed a significant positive correlation between the mean change in TT in the varicocelectomy group (+1.18 ng/mL) and testicular size (+1.508 cc) improvement. This correlation was not found in the control group: the mean TT change was −0.0748 ng/mL, and the mean change in testicular size was −0.798 cc (*P* = 0.005). The TT normalized in 77% of the varicocelectomy patients with hypogonadism. In our study we noted a significant normalization of TT values due to the marginal lower TT levels before varicocelectomy as compared to other studies which shows the importance of early diagnosis and surgical treatment. Few previous researchers have reported the relationship between varicocele and hypogonadism [31, 32]. Younes [31] reported on 48 impotent men who underwent bilateral varicocelectomy. He reported that the serum testosterone level was significantly increased in impotence and male infertility compared to levels achieved in fertile groups. He also reported that the improvement of sexual activity was 50–70%. 

Hypoactive sexual desire was found in 144/200 (72%) of men, and premature ejaculation was observed in 31/200 (15.5%). Lotti and coworkers [33] elegantly analyzed the impact of varicocele on sexual function in a large series of subjects. It is important to note the negative impact of infertility on sexual desire [34–38]. Smith et al. [38] reported that male factor infertility is an important clinical significant predictor of sexual and personal strain independent of age, race, religion, household income, educational level, and prior fertility characteristics. Moreover, Drosdzol and Skrzypulec [39] pointed out that diagnosed male factor and infertility duration of 3–6 years were connected with the highest relationship instability and the lowest sexual satisfaction both in female and male infertile. The infertility duration reported in the later study is similar to the median duration in the current study, 3.23 ± 1.75 years in study group. However, the significant difference in infertility duration among the study group and the control group may eliminate this factor as a cause of sexual dysfunction at baseline and in the follow-up visits. The overall frequency of ED was found to be 76 (53.9%) which is lower than what is reported by Lotti et al. [33] (94.7%). The difference is probably related to the study population itself. Their mean age was reported to be 52.0 + 12.9 years as compared to our younger participants 30.04 ± 4.9 years in the study group. 

Three patients in the study group had mild intermittent scrotal discomfort. The discomfort resolved completely after varicocelectomy. This relief was reported by other investigators [40–42]. However, we do not think that such discomfort could affect sexual activity. The reported frequency of pain and discomfort associated with varicocele is 2–10% [43]. In this study, it was 3% much same as per the literature. Because of this small number, it is difficult to discuss the possible effects on pain relief after varicocelectomy and its impact on sexual activity. 

## 5. Conclusion

Varicocelectomy improves serum testosterone level significantly in men with hypogonadism and infertility. It also improves the erectile dysfunction status of the men with hypogonadism. Further randomized controlled studies are needed to prove the favorable outcome of varicocelectomy in men with hypogonadism and infertility. 

## Figures and Tables

**Table 1:** Age distribution of patients in study and control group.

Age in years	Number of patients study group	Number of patients control group
21–30	57	51
31–40	40	45
41–50	3	4
Total	100	100

Mean ± SD: 30.04 ± 4.9—Study Group.

Mean ± SD: 31.73 ± 4.5—Control Group.

**Table 2:** Lateralization of varicocele in study and control groups.

Group	Bilateral	Left only
Study	34	66
Control	36	64

**Table 3:** Preoperative average serum testosterone levels in study and control groups.

Group	S. testosterone (ng/dL)
Study	177.2 ± 18.44
Control	184.52 ± 10.60

**Table 4:** Preoperative average testes sizes in study and control groups.

Group	Testes size (mL)
Study	8.14 ± 4.26
Control	8.76 ± 1.99

**Table 5:** Preoperative average internal spermatic vein diameters in study and control groups.

Group	Vein diameters (mm)
Study	3.79 + 0.42
Control	3.52 + 0.37

**Table 6:** Comparisons of age, infertility duration, testicular size, maximum vein diameter, sperm count, total testosterone pre- procedure between the study group and control group.

Variables	Control group	Study group	*P* value
Age (years)	31.73 ± 4.51	30.04 ± 4.91	0.012*
Duration of subfertility/infertility (years)	2.39 ± 0.98	3.23 ± 1.75	<0.001**
Total testosterone (ng/mL)	1.85 ± 0.11	1.77 ± 0.18	0.001**
Testicular size (cc)	8.76 ± 1.92	8.15 ± 3.49	0.119
Max vein diameter (mm)	3.53 ± 0.38	3.38 ± 0.64	0.046*
Semen analysis: count (millions/mL)	12.75 ± 5.80	12.18 ± 5.53	0.477

**Table 7:** Comparisons of change in testicular size, sperm count, and total testosterone levels before and after surgery among the study group and control group.

Variable		Control group	Study group	*P* value
Total testosterone (ng/mL)	Before	1.85 ± 0.11	1.77 ± 0.18	0.001**
	After	1.77 ± 0.29	3.01 ± 0.43	<0.001**
	Δ	−0.0748	+1.240	—
	*P* value	0.005**	<0.001**	—

Testicular size (cc)	Before	8.76±1.92	8.15 ± 3.49	0.119
	After	7.99±2.73	9.65 ± 3.51	<0.001**
	Δ	−0.798	+1.508	—
	*P* value	<0.001**	<0.001**	—

Sperm count (millions/mL)	Before	12.75 ± 5.80	12.18 ± 5.53	0.477
	After	12.44 ± 4.08	17.43 ± 6.17	<0.001**
	Δ	−0.285	+5.253	—
	*P* value	0.683	<0.001**	—

Δ is the difference.

Within group analysis by Student *t* test (Paired).

Between group analysis by student *t* test (Unpaired).
